# *rpoN1*, but not *rpoN2*, is required for twitching motility, natural competence, growth on nitrate, and virulence of *Ralstonia solanacearum*

**DOI:** 10.3389/fmicb.2015.00229

**Published:** 2015-03-24

**Authors:** Suvendra K. Ray, Rahul Kumar, Nemo Peeters, Christian Boucher, Stephane Genin

**Affiliations:** ^1^Department of Molecular Biology and Biotechnology, Tezpur UniversityTezpur, India; ^2^Laboratoire des Interactions Plantes-Microorganismes, INRA, UMR 441Castanet-Tolosan, France; ^3^Laboratoire des Interactions Plantes-Microorganismes, Centre National de la Recherche Scientifique, UMR 2594Castanet-Tolosan, France

**Keywords:** sigma 54, pathogenicity, bacterial wilt, alternative sigma factor, regulation, tomato, natural transformation, type IV pili

## Abstract

The plant pathogen *Ralstonia solanacearum* has two genes encoding for the sigma factor σ^54^: *rpoN1*, located in the chromosome and *rpoN2*, located in a distinct “megaplasmid” replicon. In this study, individual mutants as well as a double mutant of *rpoN* were created in *R. solanacearum* strain GMI1000 in order to determine the extent of functional overlap between these two genes. By virulence assay we observed that *rpoN1* is required for virulence whereas *rpoN2* is not. In addition *rpoN1* controls other important functions such twitching motility, natural transformation and growth on nitrate, unlike *rpoN2*. The *rpoN1* and *rpoN2* genes have different expression pattern, the expression of *rpoN1* being constitutive whereas *rpoN2* expression is induced in minimal medium and in the presence of plant cells. Moreover, the expression of *rpoN2* is dependent upon *rpoN1*. Our work therefore reveals that the two *rpoN* genes are not functionally redundant in *R. solanacearum*. A list of potential σ^54^ targets was identified in the *R. solanacearum* genome and suggests that multiple traits are under the control of these regulators. Based on these findings, we provide a model describing the functional connection between RpoN1 and the PehR pathogenicity regulator and their dual role in the control of several *R. solanacearum* virulence determinants.

## Introduction

*Ralstonia solanacearum* is a plant pathogenic bacterium that causes a lethal wilt disease in more than 200 plant species including many plants of economical interest such as tomato, potato, eggplant, banana, groundnut, or trees like Eucalyptus. The bacterium lives in two separate niches: in the soil as a saprophyte and inside the plant as a parasite (Genin, 2010). To coordinate the change from a saprophytic to a parasitic mode of living, an elaborate sensory network is used by the bacterium to regulate the expression of virulence and pathogenicity genes (Schell, 2000; Genin and Denny, 2012). The regulation enables growth and spread of the bacterium inside the host plant by overcoming the plant defense response. A hallmark of pathogenicity gene regulation in *R. solanacearum* is the interaction among different two component regulation systems (TCRSs) present in this bacterium (Schell, 2000). The TCRSs characterized in this bacterium are Phc, Prh, Peh, Sol, and Vsr systems (Yoshimochi et al., 2009; Genin and Denny, 2012). The TCRSs perceive signals from the environment and regulate expression of multiple pathogenicity factors. The interplay of the regulatory components of this intricate regulatory network is still poorly understood and the nature of several inductive environmental signals is unknown (Schell, 2000; Genin and Denny, 2012; Zuluaga et al., 2013). For example, the PehSR system has been demonstrated for its role in twitching motility, flagellar motility, production of polygalacturonases, and virulence (Allen et al., 1997; Kang et al., 2002). But the signaling molecule that activates PehS (sensor kinase) is unknown and the mechanism of PehR (response regulator) regulation of the downstream genes is yet to be demonstrated.

*R. solanacearum* has a bipartite genome that comprises a chromosome of 3.7 Mb and a second replicon called a megaplasmid of 2.1 Mb (Salanoubat et al., 2002). Important pathogenicity genes are distributed both on the chromosome and the megaplasmid. For example, genes encoding the type III secretion system are megaplasmid-borne whereas the *phcA*-*phcR*-*S* and *vsrA-D, vsrB-C* virulence regulatory genes are located on the chromosome. It has also been shown that paralogous gene families can be distributed on both replicons as in the case of the GALA (*ripG*) effector family members (Remigi et al., 2011). There is also evidence of apparent gene duplications on the two replicons as observed for some genes encoding Type III effector proteins (*ripAX* family), structural components such as the Flp pilus (Wairuri et al., 2012), hemagglutinins, several metabolic enzymes (including glutamine synthetase, and sarcosine oxidase), and regulatory genes. One example of the latter class is *rpoN*, which encodes the sigma factor σ^54^ (Sigma-54). Out of the two *rpoN* genes, the *rpoN1* (RSc0408) is present in the chromosome and *rpoN2* (RSp1671) is present in the megaplasmid. The role of *rpoN* in *R. solanacearum* virulence is not known.

Though *rpoN* gene is historically known for its role in nitrogen assimilation, the gene has also been shown to be involved in regulating other important functions in bacteria (Kohler et al., 1989). *rpoN* has been demonstrated to regulate multiple determinants in diverse pathogenic bacteria such as type I and type IV pili, flagellar motility, type III and type VI protein secretion systems, biosynthesis of exopolysaccharides or biofilm formation (O'Toole et al., 1997; Reitzer and Schneider, 2001; Kazmierczak et al., 2005; da Silva Neto et al., 2008; Dong and Mekalanos, 2012; Hao et al., 2013). A recent comprehensive computational study of different Sigma-54 interacting activators in bacteria indicated that Sigma-54 regulates processes that involve physical interaction of an organism with its environment like host colonization or biofilm formation (Francke et al., 2011).

In this study, we report the involvement of *rpoN1* in virulence, twitching motility, natural transformation and growth on nitrate in *R. solanacearum* GMI1000 strain, which are functions not fulfilled by *rpoN2*. Our study therefore reveals that the function of the two paralogous *rpoN* proteins is not redundant in *R. solanacearum*.

## Materials and methods

### Bacterial strains and growth conditions

The relevant characteristics of the plasmids and bacterial strains used in this work are listed in Table 1. *Escherichia coli* strains were grown at 37°C in Luria-Bertani medium (Ausubel et al., 1989). *R. solanacearum* strains were grown in complete BG medium or in MP minimal medium supplemented with glucose (5 g L^−1^ at final concentration). The composition of BG medium (Plener et al., 2010) is as follows (g L^−1^): Bacto peptone, 10; Casamino Acids, 1; yeast extract, 1. BG medium was supplemented with glucose (5 g L^−1^) and triphenyltetrazolium chloride (0.05 g L^−1^) for agar (15 g L^−1^) plates. The composition of MP medium (Plener et al., 2010) is as follows (g L^−1^): FeSO_4_–7H_2_O, 1.25 × 10^−4^; (NH_4_)_2_SO_4_, 0.5; MgSO_4_–7H_2_O, 0.05; KH_2_PO_4_, 3.4. The pH was adjusted to 7.0 with KOH. When needed, antibiotics were added to the media at the following final concentrations (mg L^−1^): kanamycin, 50; spectinomycin, 40 for *R. solanacearum*; gentamicin, 10; tetracycline, 10; ampicillin, 100 for *E. coli*.

**Table 1:** **List of plasmids, *R. solanacearum*, and *E. coli* strains used in this study**.

**Plasmid/Strain**	**Genotype**	**Antibiotic**	**References**
**PLASMIDS**			
pGEMT	Cloning vector	Ap	Promega
pCZ367	Insertional vector with *lacZ* reporter	Ap, Gm	Cunnac et al., 2004
pGRS595	pGEM-T with Ω Km insertion in *rpoN1* gene	Km	This work
pGRS596	pGEM-T with Ω Spc insertion in *RSc0407* gene	Spc	This work
pGRS597	pGEM-T with Ω Spc insertion in *pehR* gene	Spc	This work
pGRS598	pGEM-T with Ω Km insertion in *pehR* gene	Kan	This work
pGRS599	pGEM-T with Ω Spc insertion in *rpoN2* gene	Spc	This work
pGRS601	pCZ367::*rpoN1*	Gm	This work
pGRS602	pCZ367::*rpoN2*	Gm	This work
***E. COLI***			
DH5α	F^−^ *recA lacZ*ΔM15		Life Technologies
***R. SOLANACEARUM***			
GMI1000	Wild-type strain		Salanoubat et al., 2002
GMI1605	*phcA::*Ω	Spc	Genin et al., 2005
GMI1750	*pilA*::pTOK2	Tc	Kang et al., 2002
GRS552	*rpoN1::Tn5*	Km	This work
GRS553	*rpoN1::*Ω	Km	This work
GRS554	*rpoN2::*Ω	Spc	This work
GRS555	*rpoN2::*Ω *rpoN1::Tn5*	Spc, Km	This work
GRS556	*RSc0407::*Ω	Spc	This work
GRS557	*RSc0407::*Ω *rpoN1::*Ω Km	Spc, Km	This work
GRS561	*rpoN2::lacZ*	Gm	This work
GRS562	*rpoN2::lacZ rpoN1::Tn5*	Gm, Km	This work
GRS566	*rpoN1::lacZ*	Gm	This work
GRS567	*pehR::*Ω	Spc	This work
GRS568	*pehR::*Ω *rpoN1::Tn5*	Spc, Km	This work
GRS569	*phcA::*Ω *rpoN1::Tn5*	Spc, Km	This work
GRS570	*phcA::*Ω *pehR::*Ω Km	Spc, Km	This work

*Ap, ampicilin resistance; Spc, spectinomycin resistance; Km, kanamycin resistance; Gm, gentamycin resistance*.

To find out if bacterial strains were capable of utilizing nitrate as nitrogen source, bacterial strains were streaked on MP minimal medium plates without (NH_4_)_2_SO_4_ but supplemented with KNO_3_ (500 μM). Difference in growth of bacterial strains having the ability to use nitrate and not having the ability to use nitrate was distinctly observed visually after 48 h of incubation.

The co-cultivation of bacteria with Arabidopsis plant cells was performed as described by Marenda et al. (1998). After growth in BG medium, bacteria were pelleted, washed and resuspended in sterile water. Hundred microliters of these suspensions, adjusted to an OD_600_ of 0.3, were inoculated into 10 ml of Arabidopsis cell cultures grown to a density of 40 g L^−1^ fresh cells. The co-cultures were maintained for 12 h at 28°C on a rotary shaker. The mixture was filtered on 20 μm pore size nylon membrane to separate bacteria and plant cells. The bacteria were then collected by centrifugation for β-galactosidase assays.

### DNA manipulations and genetic constructs

Standard recombinant DNA techniques were performed as described previously (Ausubel et al., 1989). Restriction enzymes, DNA ligase, and other DNA enzymes were used according to the manufacturers' recommendations. Standard PCR reactions were set up with the following reagents: 4 μL of 5X GoTaq buffer (Promega, Madison, WI), 0.6 μL DMSO (5%), 0.4 μL dNTP (10 mM), 1 μL each primer (10 μM), 0.1 μL Taq (5U/μL), template DNA 20 ng, then the volume adjusted to 20 μL with deionized water.

*lacZ* reporter fusions with *rpoN1* and *rpoN2* were created by cloning DNA fragments of these two genes into the pCZ367 integrative vector (Cunnac et al., 2004). These constructs (Table 1) were introduced by transformation in strain GMI1000 as already described (Cunnac et al., 2004).

### Creation of disruption mutants for the *rpoN1, rpoN2, pehR*, and RSc0407 genes

Disruption mutants were created with the use of the Ω interposon carrying resistance gene against either Spectinomycin or Kanamycin (Prentki and Krisch, 1984). DNA fragments encompassing the target open reading frames were PCR-amplified into the pGEMT vector. The restriction sites used for cloning the interposon and their position within the target coding sequence is shown in Supplementary Material Figure 1A and the list of primers used to amplify the gene coding sequences from the GMI1000 genome is provided in Supplementary Figure 1B. The obtained plasmids (see Table 1) were linearized and used to transform *R. solanacearum* as described below. Double recombination events were selected using appropriate antibiotic resistance and checked by PCR. Insertion of the Ω interposon in the *rpoN1, rpoN2, pehR*, RSc0887 gene sequences was confirmed by PCR amplification using one oligonucleotide specific to the interposon (5′-TGTTACCCGAGAGCTTG-3′) and a second one specific to the target gene.

The *rpoN*::EZ-Tn5™ strain was obtained from the *R. solanacearum* GMI1000 library as described by Plener et al. (2012) and available at the following web address: http://iant.toulouse.inra.fr//bacteria/annotation/site/prj/ralso/tools/mutants_db/cgi/EZLucene.cgi. Insertion and fine mapping of Tn*5* in the *rpoN1* gene was confirmed by the PCR using primers against the supposed flanking regions of EZ-Tn5™ insertion (Supplementary Material Figure 1A).

### Procedure for *R. solanacearum* transformation

The protocol used followed the method described by González et al. (2011): *Ralstonia solanacearum* was grown in MP medium supplemented with glycerol (20 g L^−1^) as sole carbon source to reach an OD600 between 0.5 and 1.0. Fifteen microliters of bacterial suspension was mixed with 3–5 μ g of linearized plasmid DNA and the mixture was deposited on a 0.45 mm cellulose nitrate filter unit placed on a BG agar medium. After 48 h of incubation at 28°C, bacteria were collected and spread on selection plates supplemented with the appropriate antibiotic.

### Twitching motility assay

The procedure followed the one described by Liu et al. (2001). Briefly, saturated culture of bacteria was diluted in BG medium 10^4^-fold. Ten microliters of the diluted culture was placed on BG-agar plate. The plates were kept covered, left at 28°C for 24 h growth and observed under a light microscope (Nikon Labophot) equipped with 5X objective.

### Swimming motility assay

Swimming motility test for *R. solanacearum* strains was done of soft agar plates following the procedure described by Tans-Kersten et al. (2004). Soft agar BG medium plates were prepared with 0.15 and 0.2% agar. Due to the low agar concentration, the medium reaches a semi-solid state on plate after 30 min. The surface of the medium was just touched by the tip of the toothpick dipped in a saturated culture of the bacterium. Plates were incubated at 28°C. Bacteria proficient for swimming motility moves radially in all directions. A white radial zone distinctly observed can be measured. The semi-solid state of the soft-agar medium is stable for even 3 days. Motility proficient bacteria can cover the whole plate. In this study, swimming motility was estimated by the measurement of the radial zone after 24 h of incubation.

### Plant pathogenicity assays

Virulence assays were done by soil drenching method. Inoculations were performed by watering 4-week-old tomato plants (*Lycopersicum esculentum* cv. Marmande) with 50 ml of a suspension containing 10^7^ CFU ml^−1^. Disease development was scored daily by using a disease index scale ranging from 0 for no symptoms to 4 for completely wilted plants. Twelve plants were inoculated for each strain in at least three independent experiments. In order to analyse the data using the Kaplan–Meier representation and the non-parametric log rank test to assess the difference of the survival curves, the data was transformed as follows: all disease index lower than 2 were considered as “0” and all disease index equal of greater than 2 were considered as “1.” Statistical analysis of the results was conducted as previously described (Remigi et al., 2011). The log rank test was used to perform between-group comparisons, testing the equivalence of the Kaplan–Meier survival curves for a pair of groups. When *p* < 0.05 the survival curves were considered as significantly different.

### β-Galactosidase assays

β-Galactosidase assays were carried out as described by Miller (1972) with the following the modifications of Genin et al. (1992): 0.15–0.25 mL of bacterial suspension was added to Z buffer to a final volume of 0.75 mL. Cells were permeabilized with 100 μL of chloroform and 50 μL of sodium dodecyl sulfate (0.1%). The reaction was started by adding 150 μL of ONPG (4 mg/mL) and stopped by addition of 375 μL of Na_2_CO_3_ (1 M). β-Galactosidase activity was expressed in Miller units.

### PatScan analysis

Genome of strain GMI1000 was scanned for the occurrence of the following RpoN-binding site consensus TGGCAC(A, G)NNNNTTGC(A, T), with one mismatch allowed, using the PatScan tool (Dsouza et al., 1997) on the *R. solanacearum* GMI1000 website https://iant.toulouse.inra.fr//bacteria/annotation/cgi/ralso.cgi.

## Results

### Two conserved *rpoN* genes in the *R. solanacearum* genome

The *rpoN1* and *rpoN2* coding sequences in strain GMI1000 have a moderate but significant relatedness (Blast2P: 44% identity; 62% similarity across the whole protein sequence). Both genes have a high G+C% (*rpoN1*: 63.80; *rpoN2*: 69.19) and are also similar with respect of their codon usage. BlastN analysis with individual coding sequences of the two genes revealed the presence of *rpoN1* homologs in chromosomes and the presence of *rpoN2* homologs in the megaplasmid in all the other tested *R. solanacearum* strains. Homologs of the two genes were also present in some other bacteria that were phylogenetically close to *R. solanacearum* (Table 2). However, *Ralstonia eutropha, Cupriavidus taïwanensis*, and *Cupriavidus necator* species have an *rpoN1* homolog but no *rpoN2* homolog. To get an insight into the evolutionary significance of *rpoN1* and *rpoN2*, we compared the level of nucleotide identity for the two genes among representative strains of the *R. solanacearum* species complex and in some phylogenetically close bacteria. This analysis revealed that the identity level among these different strains was similar for *rpoN1* and *rpoN2* and followed a pattern comparable to the one of *rpoD*, encoding the essential sigma factor Sigma-70, which was used as control (Table 2). The nucleotide variation among the orthologous sequences in different strains is in agreement with the established species phylogeny which places phylotype I strains as more closely related to phylotype III than phylotype II or IV strains (Prior and Fegan, 2005). This observation suggests that *rpoN2* arose in *R. solanacearum* from an ancient gene duplication or acquisition, prior to the phylotype divergence. The apparent conservation of identity for both genes also suggested that the evolutionary selection to maintain *rpoN1* and *rpoN2* genes has been similar in the different *R. solanacearum* strains.

**Table 2:** **Relatedness of the *rpoN1, rpoN2*, and *rpoD* genes with homologs within the *R. solanacearum* species complex and other β-Proteobacteria**.

***R. solanacearum* strain or related species**	***R. solanacearum* phylotype**	**Nucleotide identity (%)**		
		***rpoN1***	***rpoN2***	***rpoD***
*R. sol*. GMI1000	I	100	100	100
*R. sol*. FQY4	I	99	99	99
*R. sol*. CMR15	III	98	98	98
*R. sol*. Psi07	IV	95	95	95
*R. syzygii* R24	IV	95	95	95
Blood disease bacterium R229	IV	95	95	94
*R. sol*. CFBP2957	II	95	93	97
*R. sol*. Po82	II	95	94	97
*R. sol*. IPO1609	II	95	93	97
*R. picketti* 12J	–	87	84	90
*R. eutropha*	–	80	absent	89
*Cupriavidus taiwanesis*	–	80	absent	88
*Cupriavidus necator*	–	79	absent	88

*Identity score was determined using the BlastN program*.

### The *rpoN1* mutant is deficient for natural transformation

Mutant strains for the *rpoN1* and *rpoN2* genes were generated. The *rpoN2*::Ω mutant was created by insertion of the Ω interposon as described previously (Plener et al., 2012). The *rpoN1* insertion mutant was obtained from an already available EZ-Tn5™ mutant library in GMI1000 (Boucher and Genin, unpublished data). In this work we used the following mutants: an *rpoN1::*EZ-Tn5 mutant (Kan*^r^*) and a *rpoN2*::Ω Sp mutant (Spc^r^).

**Figure 1:**
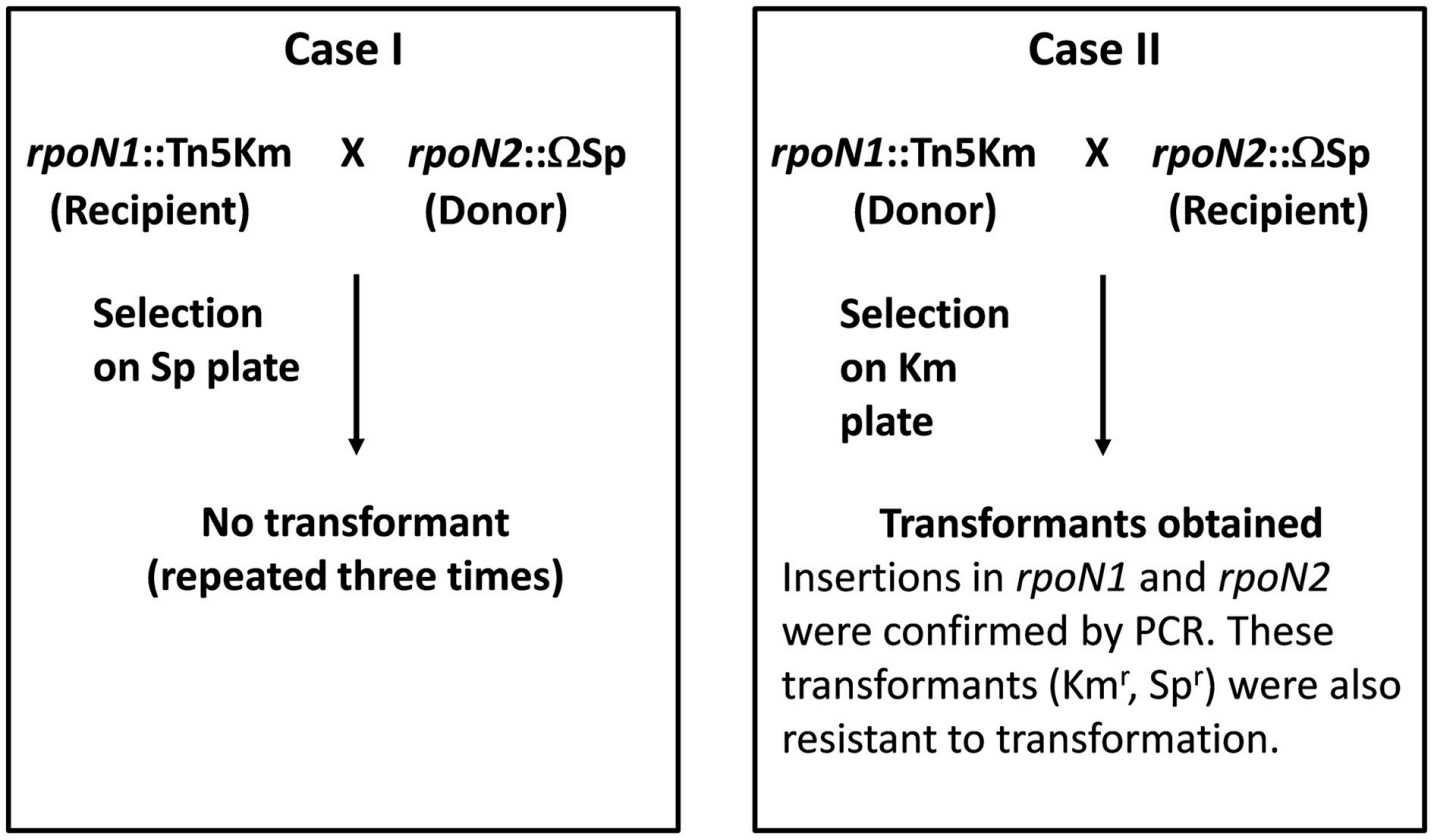
**Genetic crosses in *R. solanacearum* GMI1000 to obtain an *rpoN1*/*rpoN2* double mutant**. Two transformation experiments were set up to obtain the *rpoN1/rpoN2* double mutants using either the *rpoN1*::Tn*5* or the *rpoN2*::Ω as receptor strains. Transformation was successful only in case II.

To create a double mutant, two experiments were set up (Figure 1). First, genomic DNA from *rpoN2*::Ω strain was used to transform *rpoN1*::EZ-Tn5 strain. Second, genomic DNA from *rpoN1*::EZ-Tn5 strain was used to transform *rpoN2*::Ω strain. Appropriate antibiotic medium was used to select for transformants. Surprisingly, transformants were only obtained in the second transformation experiment but not from the first transformation experiment in spite of repeating the experiment several times. As we could obtain the *rpoN1 rpoN2* double mutant (*rpoN1*::EZ-Tn5 *rpoN*2::Ω; hereafter referred to *rpoN1/2*) in the second transformation experiment, the inability to get transformation in *rpoN1*::EZ-Tn5 background indicated that the GMI1000 strain with the genotype *rpoN1*::EZ-Tn5 was likely inefficient or strongly affected for transformation. In a separate experiment, mutation in a different gene [RSc3392::Ω ] also failed to transfer into the *rpoN1*::EZ-Tn5 background. This eliminated the possibility that the *rpoN2* mutation was lethal in the *rpoN1*::EZ-Tn5 background and further supported the view that the *rpoN1*::EZ-Tn5 strain was inefficient for transformation. Any negative impact of EZ-Tn5 itself on transformation can be ruled out because three independent EZ-Tn5 mutants of GMI1000 strains were able to undergo transformation with *rpoN2*::Ω as recipient strain. Further, independent transformants could be obtained in GMI1000 using genomic DNA from the *rpoN1*::EZ-Tn5 strain. These new mutants were also resistant to transformation which eliminated the possible effect of any mutation at a distant locus in the earlier strain.

To eliminate the possibility of any tightly linked locus to *rpoN1*::EZ-Tn5 responsible for this transformation resistant phenotype, a new *rpoN1* mutant was created by insertion of the Ω interposon [*rpoN1*::Ω (Km)] (Supplementary Material Figure 1) and this new mutant was also found to be transformation deficient. These genetic experiments confirmed that insertion mutation in *rpoN1* abolished natural transformation ability in GMI1000 whereas insertion mutation in *rpoN2* had no effect. As expected the *rpoN1*/*2* double mutant was also inefficient for transformation.

A survey of the *rpoN1* locus indicated that there is an ORF (RSc0407) just downstream *rpoN1* and which presumably belongs to the same transcriptional unit (see https://iant.toulouse.inra.fr/bacteria/annotation/cgi/ralso.cg). A disruption of the RSc0407 gene was created (RSc0407::Ω) and proved that this *rpoN1* downstream gene had no effect on *R. solanacearum* transformation. Altogether, these results showed that *rpoN1* is required for natural competence in *R. solanacearum*.

### *rpoN1* is required for twitching motility and growth on nitrate

**Figure 2:**
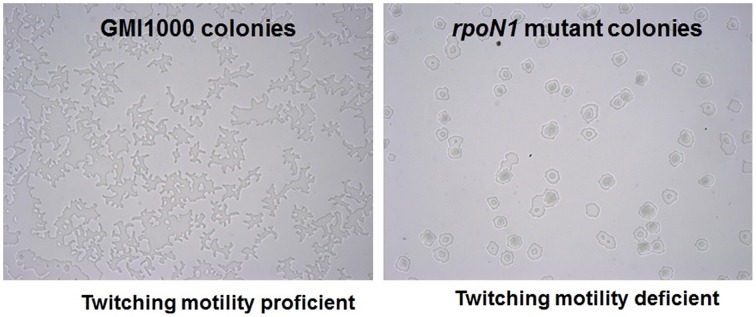
***rpoN1* mutants are deficient in twitching motility**. GMI1000 and its *rpoN1* mutant derivative were checked for twitching motility under a light microscope. GMI1000 was proficient for twitching motility as evidenced by the typical finger-like projections coming out from the colony (left) whereas the *rpoN1* mutant strain was formed circular shaped colonies (right).

There is no previous report in any bacteria relating the role of *rpoN* in natural transformation. However, it has been demonstrated that type IV pili mutants of *R. solanacearum* are deficient for transformation as well as for twitching motility (Kang et al., 2002). In addition, there is a potential *rpoN* promoter sequence in the upstream region of the *pilA* gene in GMI1000 genome and *pilA* is required for the formation of type IV pili (Kang et al., 2002). It has also been reported that *rpoN* is involved in the formation of type IV pili and therefore twitching motility in *Pseudomonas aeruginosa* (Ishimoto and Lory, 1989; Semmler et al., 1999) and *Neisseria elongata* (Rendón et al., 2013). We thus studied twitching motility in *rpoN1, rpoN2* and the *rpoN1/2* mutants. We observed that the *rpoN1* and *rpoN1/2* mutants were twitching motility-deficient whereas the *rpoN2* mutant was proficient for twitching motility as the wild type strain (Figure 2). This indicated that *rpoN1* mutant is likely to be type IV pili deficient because type IV pili are required for twitching motility in *R. solanacearum*. The RSc0407::Ω mutation had no impact on twitching motility, thus confirming that the *rpoN1* mutation had no polar effect on the downstream gene.

The initial discovery of the *rpoN* gene in bacteria was related to its role in nitrogen assimilation (Buck et al., 2000). We studied growth of different *R. solanacearum* strains on 500 μM nitrate. The *rpoN1* as well as *rpoN1/2* mutants did not grow when nitrate was supplied as the only nitrogen source while *rpoN2* and RSc0407::Ω mutants exhibited growth similar to GMI1000 in this medium (Supplementary Material Figure 2).

### *rpoN1* mutants are impaired in virulence on tomato plants

**Figure 3:**
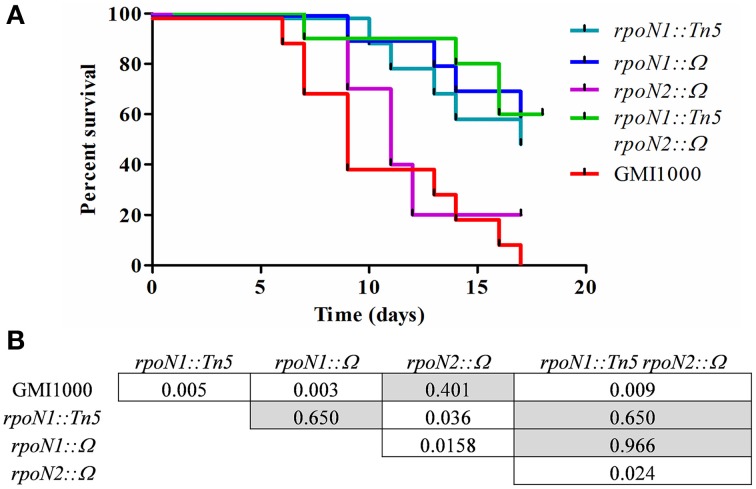
***rpoN1* mutants are impaired in virulence. (A)** Kaplan–Meier survival analysis of tomato plants inoculated with *R. solanacearum* GMI1000 (wild-type) and mutant derivatives. Each strain was inoculated on 12 tomato plants in three independent experiments. **(B)** *P*-values from Gehan–Breslow–Wilcoxon tests are associated with each graph. *p*-value of <0.05 are indicated in white boxes.

To understand the role of *rpoN1* and *rpoN2* in *R. solanacearum* virulence, we independently inoculated tomato plants with *rpoN1, rpoN2, rpoN1/2*, RSc0407::Ω mutants and compared their virulence phenotype with the wild type GMI1000 by a soil drenching inoculation method (Figure 3). A Kaplan–Meier survival analysis shown in Figure 3A revealed that the *rpoN1* and *rpoN1/2* mutants were strongly reduced in pathogenicity. A log-rank test, aimed to verify the hypothesis of similarity of the survival curves, confirmed that the difference observed with the *rpoN1* mutant, but not the *rpoN2* mutant, was statistically significant (Figure 3B). The virulence deficiency observed in *rpoN1* and as well as in *rpoN1/2* double mutants could be expected because it was previously reported that twitching motility is required for full virulence of *R. solanacearum* (Kang et al., 2002), which is in agreement with the observed *rpoN1* and *rpoN1/2* twitching motility-deficient phenotype.

The growth proficiency of *rpoN1* mutants was compared with the GMI1000 wild-type strain in complete medium. The generation time of both *rpoN1* mutant strains and GMI1000 was similar (i.e., 2.0 h). On medium without antibiotic *rpoN1* mutant and GMI1000 strains formed small and big colonies, respectively, which can be easily observed after 2 days of growth on solid medium (Supplementary Material Figure 3A). This small colony phenotype presumably resulted from the twitching motility-deficiency of the *rpoN1* mutant and suggested that the virulence deficiency *rpoN1* is not due to growth deficiency.

### The *rpoN1* mutant phenocopies the *pehR* mutant for twitching motility

To understand any other reason apart from the twitching motility responsible for the virulence deficiency in *rpoN1* mutants, we decided to compare it with the *pehR* mutant, since *pehR* is a component of the virulence regulatory network in *R. solanacearum* (Allen et al., 1997) which is also deficient for twitching motility (Tans-Kersten et al., 2004). We constructed a *pehR* disruption mutant in strain GMI1000 and compared it to the *rpoN1* mutant with respect to twitching motility. This *pehR* mutant was also deficient for twitching motility (Supplementary Material Figure 3B). Virulence deficiency observed for the *rpoN1* and *pehR* mutants was also found to be of similar magnitude in soil drench inoculation assay (Supplementary Material Figure 4). *rpoN1* and *pehR* mutants were both deficient for twitching motility but the *pehR* mutant strain grows on nitrate unlike *rpoN1*.

*pehR* mutants are deficient for swimming motility (Allen et al., 1997). In complete medium GMI1000 exhibits no motility so we took advantage of the *phcA* regulatory mutant that expresses constitutive motility (Brumbley et al., 1993). To compare the role of *rpoN1* and *pehR* in *R. solanacearum* swimming motility, we therefore created *phcA*/*pehR* and *phcA*/*rpoN1* double mutants. Swimming motility assays were performed on semi-solid agar plates and motility of the strains was compared based on the measurement of the bacterial halo diameter formed after 24 h (Supplementary Material Table 1). This assay revealed that the *pehR* mutation, but not *rpoN1*, abolished the constitutive motility observed in a *phcA* mutant background. This phenotypic analysis of the *rpoN1* and *pehR* mutants supported the view that both genes are jointly involved in regulating twitching motility in *R. solanacearum* whereas *pehR* appears to regulate swimming motility independently of *rpoN1* and *rpoN1* regulates nitrate assimilation independently of *pehR*.

### *rpoN1* controls the expression of *rpoN2*

In order to identify candidate *rpoN* regulatory targets, the whole GMI1000 genome sequence was scanned with a consensus motif found in σ54-dependent promoters (TGGCACRNNNNTTGCW) (Barrios et al., 1999). A total 79 hits were found. Out of these 79 hits, 40 sites were considered as potential Sigma-54 target sequence because the consensus motif was located in the sense orientation in the 450 bp region immediately upstream of an annotated gene (Salanoubat et al., 2002). The list of the 40 identified genes is given in Table 3. In agreement with the former results, this list contains the *pilA* gene encoding the structural component of Type IV pili and the RSp1219–RSp1223 operon which was found to control nitrate assimilation in *R. solanacearum* (Dalsing and Allen, 2014). Among these 40 *rpoN*-regulated gene candidates, more than one-third encode hypothetical proteins of unknown function and six encode uncharacterized transcription regulators.

**Table 3:** **List of predicted σ^54^ factor-target sequences in the *R. solanacearum* GMI1000 genome**.

	**Gene**	**Sequence element**	**Position upstream of start codon**	**Product**
1	RSc0133	TGGCGCATTCATTGCA	73	Hypothetical
2	RSc0223^*^	TGGCACGCCCGTTGCA	94	Hypothetical
3	RSc0330	TGGCATGGCCCTTGCA	146	Dicarboxylate transporter
4	RSc0341	TGGCATGACAGTTGCA	68	Hypothetical
5	*pilA*	TGGCACGGTCCCTGCT	47	Type 4 pilin
6	RSc0731	TGGCAGGCTGTTTGCT	22	Thioesterase
7	RSc0753	TGGCGCGCGATTTGCT	162	Hypothetical
8	RSc0798^*^	TGGCAGGCAATTTGCA	34	Nucleoside permease operon
9	RSc0940	TGGCACGACTGGTGCA	10	Ribosome small unit GTPaseA
10	RSc0950	TGGCACATTTTTCGCT	19	Hypothetical
11	RSc1121	TGGCAGATATCTTGCT	377	Hypothetical
12	RSc1878	GGGCACACCGCTTGCT	100	Hypothetical
13	RSc2041	TGGCACGCTAGTTGCG	50	Transporter
14	RSc2102	TGGCACAAAACTCGCA	55	Transcription regulator
15	RSc2118^*^	TGGCCCAGCACTTGCA	178	Transporter
16	RSc2173^*^	CGGCACGGGATTTGCA	280	ABC transporter operon
17	RSc2194	TGGCACGGTCAGTGCT	54	Hypothetical
18	RSc2312	TGGCGCATTCCTTGCT	153	Transcription regulator
19	RSc2320	TGGCACAAAAGTTTCT	407	Transcription regulator
20	RSc2441	TGGCACGCTTCTTGTT	430	Transporter
21	RSc2641	TGGCACGTCGATTGCG	71	Hypothetical
22	RSc2930	CGGCACGCCTCTTGCA	79	Mechanosensory ion channel
23	RSc3128	TGGCCCGGGCCTTGCA	68	Dehydrogenase
24	RSc3203	TGGCACGCCGCTTTCA	108	Hypothetical
25	RSc3410^*^	TGGCATGCCCATTGCA	69	ABC transporter operon
26	RSp0054	TGGCCCGCTTGTTGCA	88	Transcription regulator
27	RSp0092	TGGAACAAGCTTTGCA	57	Hypothetical
28	RSp0219	TGGCACAGGGCTTGCC	157	Hydroxylase
29	RSp0229	TGGCATGGCGCTTGCA	70	Dehydrogenase
30	RSp0285	TGGCCCGCCGCTTGCT	248	Transcription regulator
31	RSp0337	TGGCACGGCTGTTGCA	94	Porin
32	RSp0635	TGGCACGCCGATTGCG	70	Acyltransferase
33	RSp0830	TGGCACACTGATTGAA	46	Galactarate dehydratase
34	RSp1093	TGGCATAGCAATTGCA	55	Hypothetical
35	RSp1094	TGGCATAGCGCTTGCA	79	Hypothetical
36	RSp1223^*^	TGGCACACCTGTTGCA	151	Nitrate assimilation operon
37	RSp1234	TGGCACGATTGTTGTT	253	Lipase
38	RSp1355	TGGCACGATTGTTGTT	253	Hypothetical
39	*rpoN2*	TGGCACAGCCTTTGCA	63	σ54-related protein
40	RSp1674	TGGCACGGCGGTTGCA	40	Hypothetical

*Genes followed by an asterisk correspond to the first gene of probable operonic gene units*.

**Figure 4:**
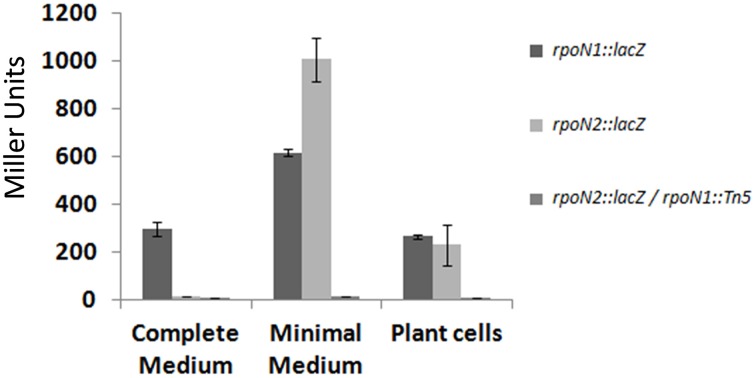
**Expression pattern of the *rpoN1* and *rpoN2* genes**. Expression study was carried out in complete and minimal media, and in the presence of Arabidopsis plant cells. The y-axis indicates β-galactosidase activity expressed in Miller units and the x-axis indicates the different conditions in which the expression study was carried out. Standard deviation was calculated from three different experiments performed independently.

Interestingly, one of the potential σ54-dependent promoter regions was found to be upstream of *rpoN2*. We studied the expression of *rpoN1* and *rpoN2* using reporter fusions with *lacZ* gene (Materials and Methods). β-galactosidase expression was monitored after cultivation of bacteria in complete and minimal media, as well as in presence of Arabidopsis plant cells. Results in Figure 4 show that *rpoN1* and *rpoN2* display a different expression pattern: *rpoN1* is significantly expressed in the three conditions tested (albeit expression appears higher in minimal medium) whereas *rpoN2* is not expressed in complete medium and is specifically induced in minimal medium. In the latter condition, the expression of *rpoN2* is completely abolished in an *rpoN1* mutant background, thus confirming that *rpoN1* regulates the expression of *rpoN2* in strain GMI1000. No specific induction of *rpoN1* and *rpoN2* expression could be observed when bacteria were co-cultivated with plant cells.

## Discussion

The role of *rpoN* (σ^54^ or also σ^N^) was historically uncovered with the study of the regulation of nitrogen metabolism but it has been subsequently found to be involved in many other biological activities in many diverse Proteobacteria (Buck et al., 2000). Evidence of *rpoN* involvement in bacterial pathogenesis and virulence, mainly through the control of flagellar motility, is also documented (Kazmierczak et al., 2005). However, *rpoN* does not share a common role in plant pathogens: in *Pseudomonas syringae, Pectobacterium carotovorum*, and *Erwinia amylovora rpoN* is required for virulence and is involved in the regulation of the type III secretion system (Hendrickson et al., 2000a,b; Chatterjee et al., 2002; Ramos et al., 2013) but in *Xanthomonas campestris* pv. *vesicatoria*, disruption of *rpoN* has no impact on virulence (Horns and Bonas, 1996). A recent study revealed that in *P. syringae rpoN* also strongly activated the vast majority of genes involved in flagellar synthesis and motility, as well as many genes activated *in planta* including phytotoxins and the siderophore pyoverdin (Yu et al., 2014).

In *R. solanacearum rpoN* mutants still induce a hypersensitive response on tobacco (data not presented) which indicates that σ^54^ is not required for the functionality of the type III secretion system. However, we show in this study that *rpoN1* significantly contributes to *R. solanacearum* pathogenesis and controls multiple traits in this bacterium. This pleiotropic phenotype was observed with two mutants carrying independent mutations in the *rpoN1* gene and further analyses showed that it was independent from the *rpoN1* downstream gene (RSc0407) (Supplementary Material Figure 3). Because two of the *rpoN1*-regulated traits (type IV pili production and nitrate assimilation) were previously found to be both required for full *R. solanacearum* virulence (Kang et al., 2002; Dalsing and Allen, 2014), the impaired pathogenicity phenotype of *rpoN1* mutants is expectable. Altogether, these reports indicate that *rpoN* gene has evolved to carry out different functions in different phytopathogens, regulating virulence determinants in some species but not in others. Interestingly, deficiency for type IV pili also leads in *R. solanacearum* to the loss of natural transformation, a property presumably important for horizontal gene transfer and the emergence of variant strains (Coupat-Goutaland et al., 2011). To our knowledge, this is the first report of natural competence as an *rpoN*-regulated trait.

**Figure 5:**
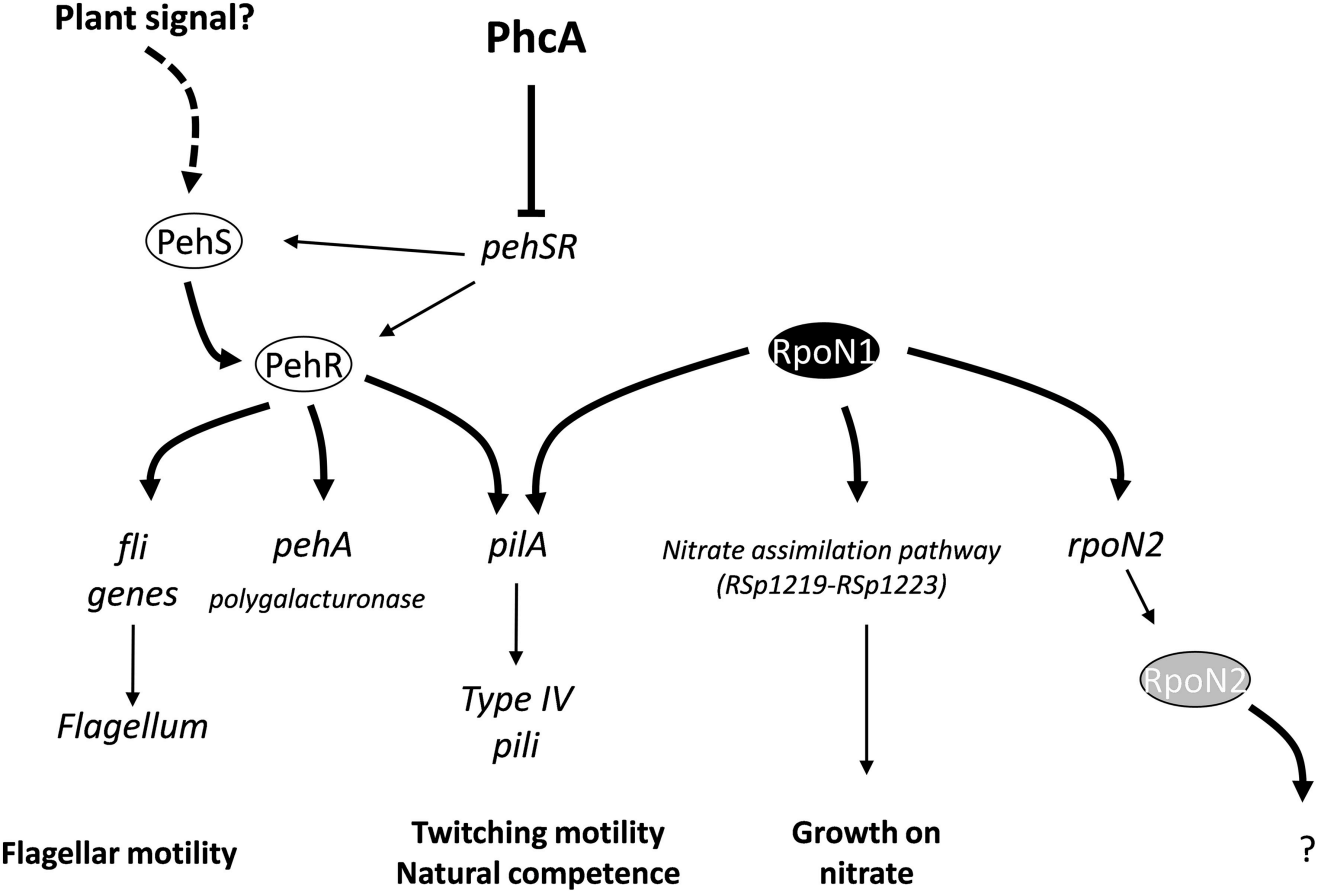
**Model describing connection between RpoN1 and PehR and their role in the control of several *R. solanacearum* virulence determinants**. Both *rpoN1* and *pehR* are necessary for expression of the *pilA* gene which encodes the Type IV pilin, a fimbrial structure required for twitching motility and natural competence. Joint binding of PehR and RpoN1 proteins on the *pilA* promoter is proposed to activate the transcription of the gene. *pehSR* genes are under the transcriptional control of the PhcA master regulator and activity of the PehS transmembrane sensor kinase is dependent upon an unknown plant signal (Allen et al., 1997).

We found that the *pehR* and *rpoN1* mutants are both required for the expression of type IV pili and twitching motility in *R. solanacearum*. Both mutants are also strongly reduced in virulence. PehR encodes a response regulator which is activated by the PehS sensor kinase (Allen et al., 1997; Tans-Kersten et al., 2004). PehS and PehR exhibit homology to the NtrB and NtrC two-component regulatory system and therefore belong to the class of bacterial enhancer binding proteins known to act as specialized activators of σ^54^-dependent transcription (Bush and Dixon, 2012). Based on these observations, we propose a model (Figure 5) in which PehR act as a σ^54^-activator protein for the control of the *pilA* gene required for the biogenesis of type IV pili. This hypothesis is supported by the finding that *pilA* contains a predicted *rpoN*-binding element and by a recent transcriptomic analysis of the *rpoN1* mutant indicating that *pilA* expression is indeed *rpoN1*-dependent (unpublished results). Our results also indicate that PehR and RpoN control some downstream genes independently from each other: flagellar motility is a PehR-regulated trait whereas nitrate assimilation is RpoN-dependent but PehR-independent. From the inventory of putative RpoN targets listed in Table 2, it is also very likely that *rpoN1* controls additional genes. For example, RSc0330, which carries an RpoN-binding element in its promoter and encodes a dicarboxylate transporter, has a homolog in *P. chlororaphis* which was shown to be under the control of *rpoN* and required for effective root colonization (Nam et al., 2006). The putative RpoN targets we identified in *R. solanacearum* GMI1000 comprise many genes of unknown function, transporters and transcription regulators, and their possible role in pathogenesis or plant colonization remains to be determined.

In most bacteria, the σ^54^ family contains just a single member. Relatively few organisms have two copies of *rpoN*, such as *Bradyrhizobium japonicum, Rhizobium etli* (Kullik et al., 1991; Michiels et al., 1998), some *Xanthomonas oryzae* pathovars (Tian et al., 2015) whereas *Rhodobacter sphaeroides* has up to four *rpoN* copies (Poggio et al., 2002). In *B. japonicum*, these copies are highly similar and functionally interchangeable (Michiels et al., 1998). In *X. oryzae* pv *oryzae rpoN2* is located close to the flagellar regulon and is required for swimming motility, having therefore a distinct role from the paralogous *rpoN1* gene (Tian et al., 2015).

Two copies of *rpoN* are generally found in β-proteobacteria having two or more replicons such as *R. solanacearum, R. picketti*, and many Burkholderiales. However, the second copy is absent in bacteria taxonomically related to *R. solanacearum* such as *R. eutropha* and *Cupriavidus* species, which are non-pathogenic. In this work we show that *rpoN1* and *rpoN2* are not functionally redundant in *R. solanacearum* since *rpoN1* is required for twitching motility, natural transformation and growth on nitrate whereas *rpoN*2 mutant is proficient for these phenotypes. We also provide genetic evidence that expression of *rpoN2* is dependent upon *rpoN1* and has a distinct expression profile, being specifically induced when bacteria are grown in minimal medium or in presence of plant cells. The *rpoN2* mutant is not altered in virulence and no specific phenotype could be associated to this second *rpoN* copy, so its role remains enigmatic. However, the broad conservation and stability of this gene in the *R. solanacearum* species complex, which suggest a long co-evolution of the two *rpoN* copies in the species, prompt us to speculate that the adaptation of the bacterium to a specific niche or environmental condition might have selected a defined regulatory role for *rpoN2* during the pathogen lifecycle. Future investigations aimed to determine the specific target promoters of *rpoN1* and *rpoN2* at the transcriptomic level will help to define the distinct roles of these two σ^54^-RNA polymerases during the interaction of *R. solanacearum* with its host plants and its environment.

### Conflict of interest statement

The authors declare that the research was conducted in the absence of any commercial or financial relationships that could be construed as a potential conflict of interest.
