# Supplementary material for: rpoN1, but not rpoN2, is required for twitching motility, natural competence, growth on nitrate, and virulence of Ralstonia solanacearum
Source: Front Microbiol. 2015 Mar 24;6:229. doi: 10.3389/fmicb.2015.00229 (PMC4371752; doi:10.3389/fmicb.2015.00229)
Supplement: Supplementary file 1 [file Table1.DOCX]

**Supplementary Material Table 1.** Motility phenotypes resulting from *pehR* and *rpoN1* mutations in a GMI1000 *phcA* mutant background.

| **Strain** | **Genotype** | **Motility (mm)*** |
| --- | --- | --- |
| GMI1605 | *phcA* | 55 ± 5 |
| GRS569 | *phcA rpoN1* | 55 ± 5 |
| GRS570 | *phcA pehR* | 25 ± 1 |

* corresponding to the halo diameter as formed after 24 h of incubation at 28°C on semi-solid 0.2% agar BG plates. Experiments were repeated three times.
