# Supplementary figures and images for: rpoN1, but not rpoN2, is required for twitching motility, natural competence, growth on nitrate, and virulence of Ralstonia solanacearum
Source: Front Microbiol. 2015 Mar 24;6:229. doi: 10.3389/fmicb.2015.00229 (PMC4371752; doi:10.3389/fmicb.2015.00229)

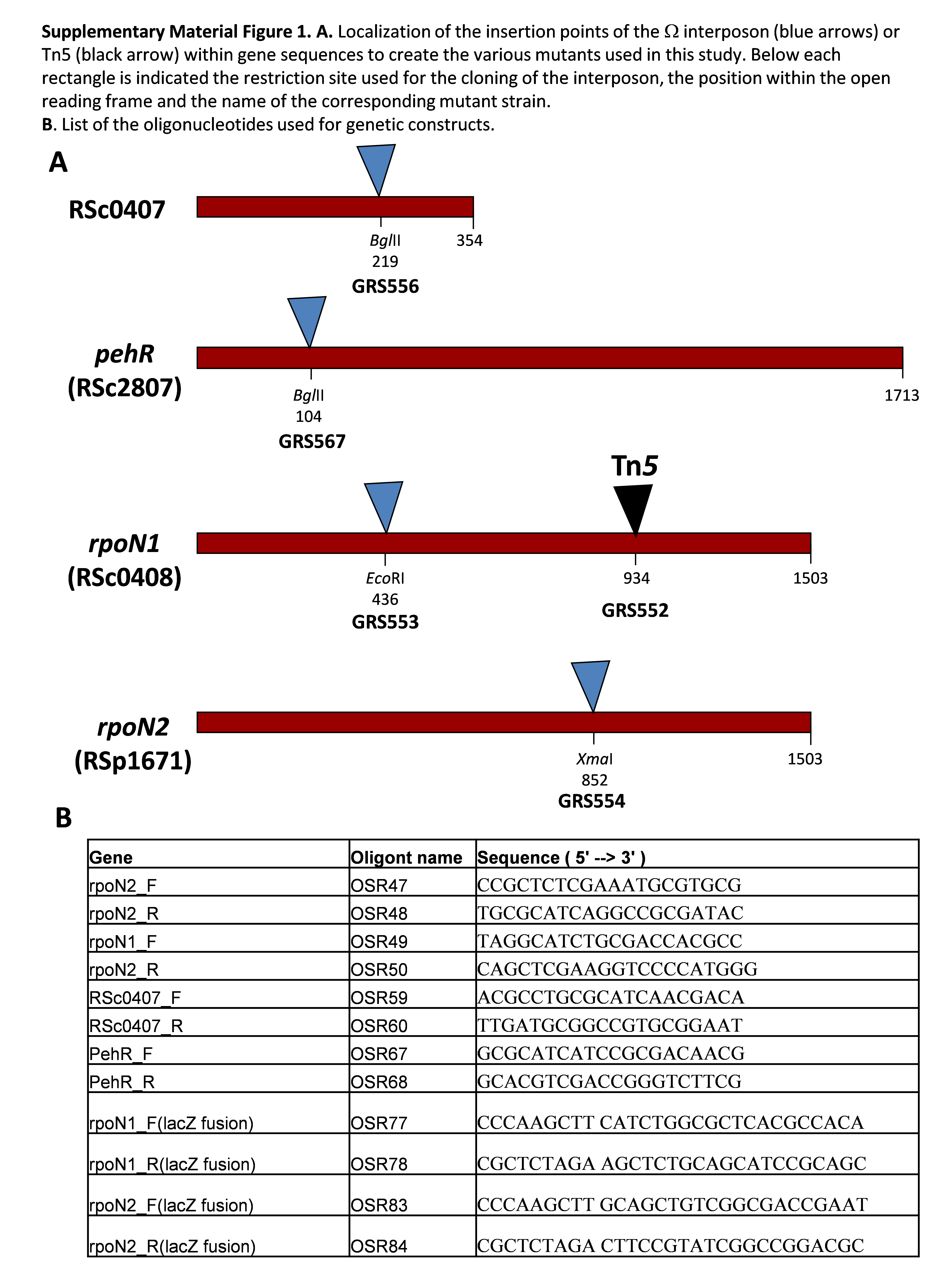

Supplement: Supplementary file 2 [file Image1.TIF]

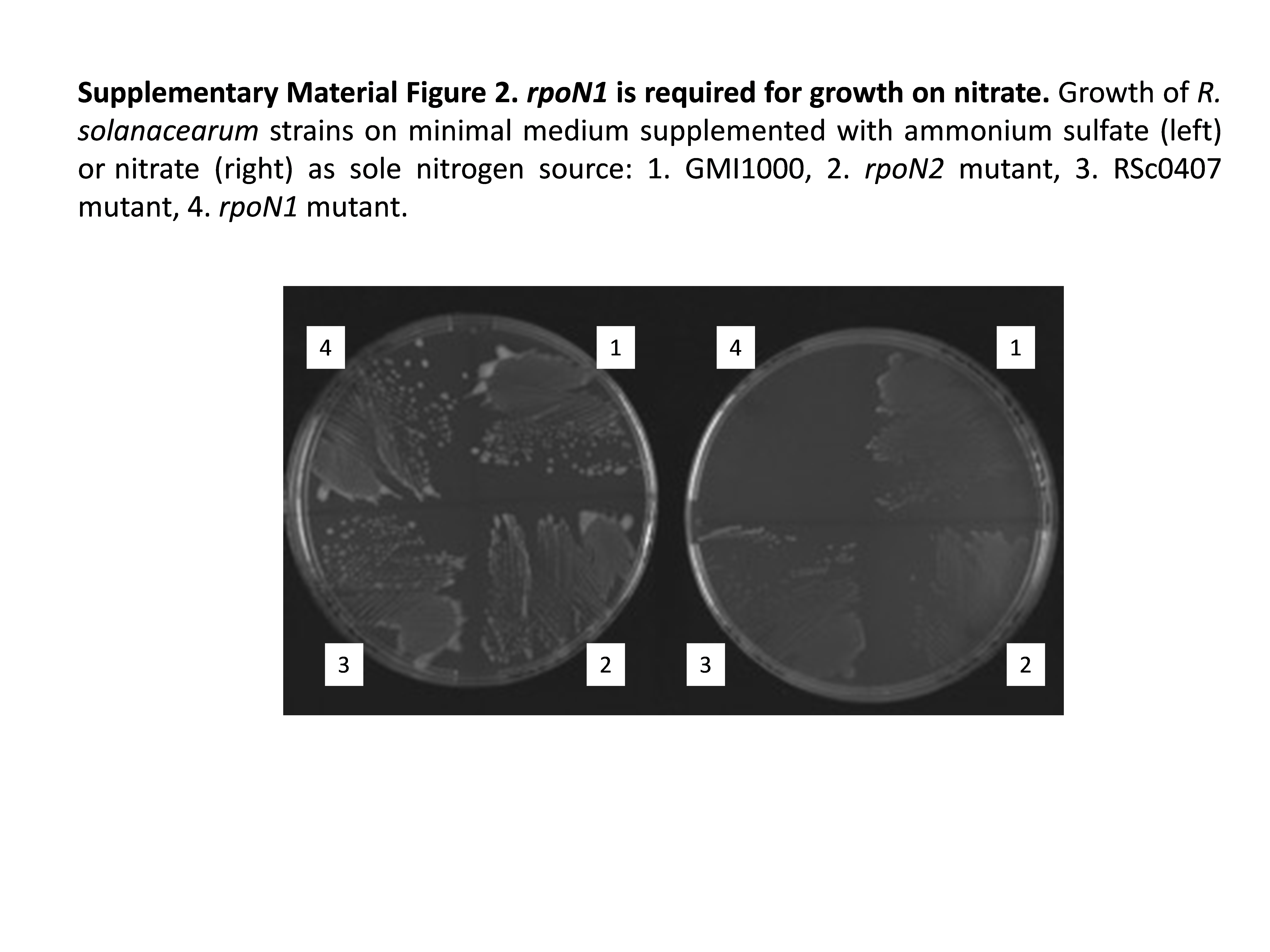

Supplement: Supplementary file 3 [file Image2.TIF]

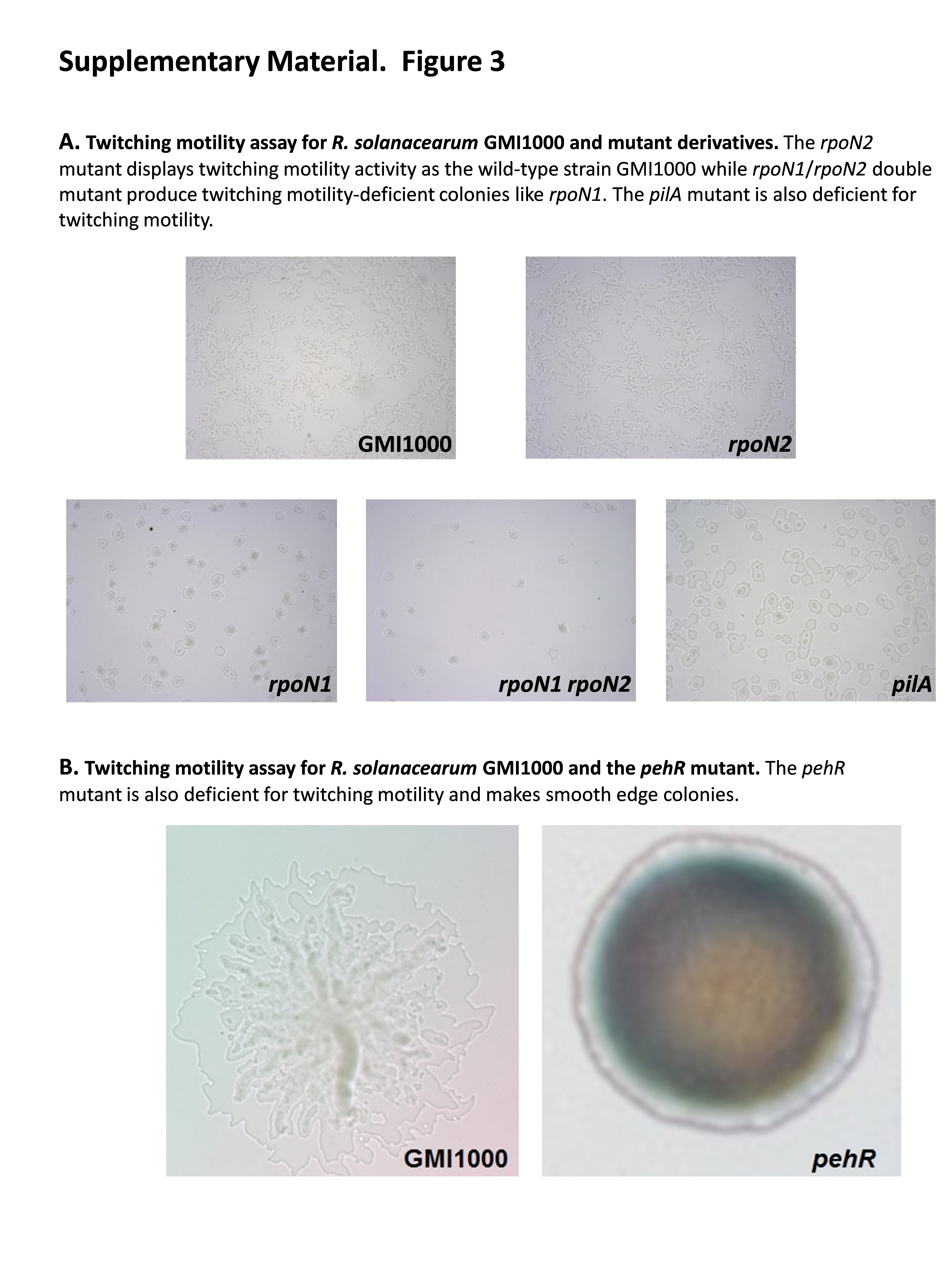

Supplement: Supplementary file 4 [file Image3.TIF]

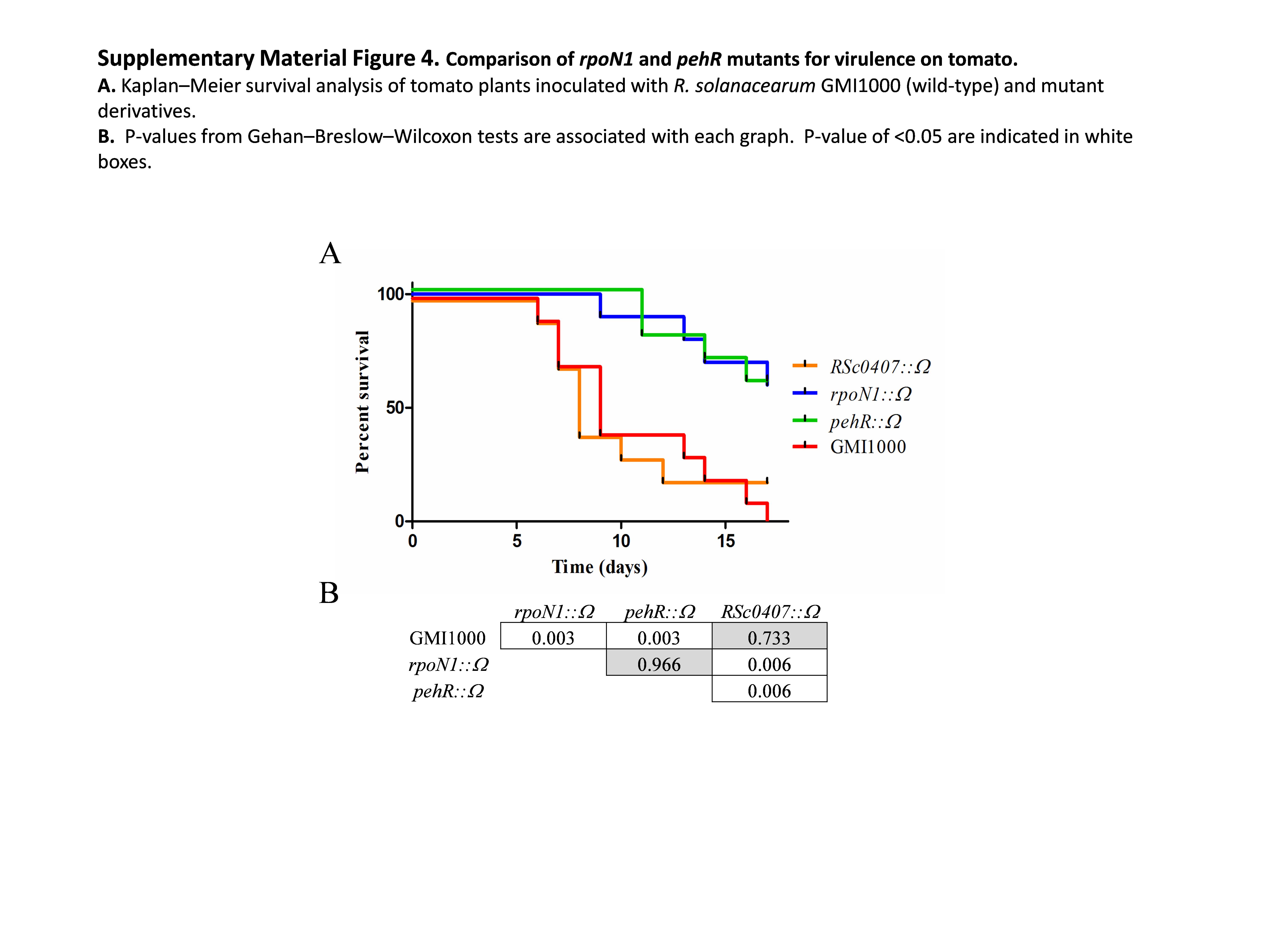

Supplement: Supplementary file 5 [file Image4.TIF]
